# Projecting the impacts of housing on temperature-related mortality in London during typical future years

**DOI:** 10.1016/j.enbuild.2021.111233

**Published:** 2021-10-15

**Authors:** Jonathon Taylor, Phil Symonds, Clare Heaviside, Zaid Chalabi, Mike Davies, Paul Wilkinson

**Affiliations:** a Department of Civil Engineering, Tampere University, Tampere, Finland; b UCL Institute for Environmental Design and Engineering, University College London, London, UK; c London School of Hygiene and Tropical Medicine, London, UK

**Keywords:** Energy efficiency, Indoor temperature, Temperature mortality, Climate change, Building physics

## Abstract

Climate change means the UK will experience warmer winters and hotter summers in the future. Concurrent energy efficiency improvements to housing may modify indoor exposures to heat or cold, while population aging may increase susceptibility to temperature-related mortality. We estimate heat and cold mortality and energy consumption in London for typical (non-extreme) future climates, given projected changes in population and housing. Building physics models are used to simulate summertime and wintertime indoor temperatures and space heating energy consumption of London dwellings for ‘baseline’ (2005–2014) and future (2030s, 2050s) periods using data from the English Housing Survey, historical weather data, and projected future weather data with temperatures representative of ‘typical’ years. Linking to population projections, we calculate future heat and cold attributable mortality and energy consumption with demolition, construction, and alternative scenarios of energy efficiency retrofit. At current retrofit rates, around 168–174 annual cold-related deaths per million population would typically be avoided by the 2050s, or 261–269 deaths per million under ambitious retrofit rates. Annual heat deaths would typically *increase* by 1 per million per year under the current retrofit rate, and 12–13 per million under ambitious rates without population adaptation to heat. During typical future summers, an estimated 38–73% of heat-related deaths can be avoided using external shutters on windows, with their effectiveness lower during hotter weather. Despite warmer winters, ambitious retrofit rates are necessary to reduce typical annual energy consumption for heating below baseline levels, assuming no improvement in heating system efficiencies. Concerns over future overheating in energy efficient housing are valid but increases in heat attributable mortality during typical and hot (but not extreme) summers are more than offset by significant reductions in cold mortality and easily mitigated using passive measures. More ambitious retrofit rates are critical to reduce energy consumption and offer co-benefits for reducing cold-related mortality.

## Graphical abstract

**Figure:**
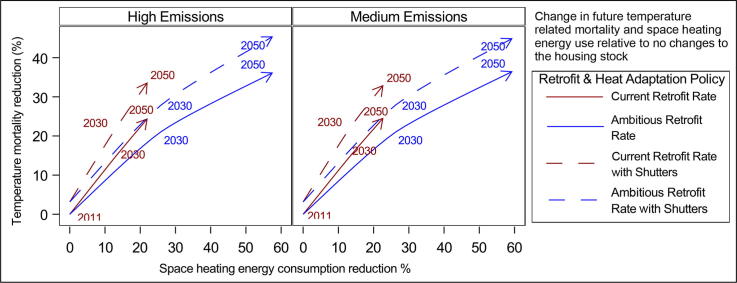

## 1 Introduction

Despite a temperate climate, weather-related mortality presents a significant health burden in the UK. Current annual temperature-related mortality is dominated by cold, associated with around 41,000 excess deaths annually in the UK [1]. The elderly are at particular risk during cold periods [1], [2], with the majority of deaths due to cardiovascular and respiratory causes [3]. In comparison, exposure to heat has a smaller impact on population mortality, with around 2000 deaths annually in the UK due to warm and hot weather [1]. As with cold, the majority of deaths from heat are from cardiovascular and respiratory causes [3], with the elderly at greater risk [1], [2], [4]. Recent heatwave events have demonstrated that high levels of mortality may occur during relatively short periods of extreme high temperatures; heatwaves in 2003, 2006, and 2018 led to 2091 [5], 680 [6], and 863 [7] excess deaths in England, respectively. However, increases in population mortality occur in England even at moderately warm temperatures [8], and in London warm (but not extreme hot) and isolated hot days are responsible for the majority of the total heat mortality burden [9]. With projected increases in temperatures and more frequent extreme heat events due to climate change [10] and an aging population, there has been an increase in research projecting how both cold and heat-related mortality may change in the future [1], [11].

An individual’s exposure to heat and cold may be modified by the buildings they inhabit, given the average English person spends 90% of their time indoors, and 70% inside their own homes [12]. The UK has some of the least energy efficient housing in Western Europe [13], and heat loss through the building fabric or through heated air escaping the building, can make housing difficult and costly to heat. Poorly insulated housing has been associated with excess winter mortality [14], while winter fuel payments (WFPs)^1^ and improvements to housing may be partly responsible for a gradual reduction in winter deaths [15]. While retrofits can help reduce winter cold exposure, increased energy efficiency is also critical for reducing energy consumption and subsequent Greenhouse Gas emissions in order to mitigate climate change. For heat, certain building characteristics such as insulation level, ventilation, and glazing may modify the risk of high indoor temperatures [16], [17], [18]. In Paris, Vandentorren et al. [19] demonstrated the importance of housing on heat-related mortality, showing an increased risk of heat mortality for occupants of top floor flats and poorly insulated dwellings.

A selection of studies have sought to estimate changes to temperature-related mortality under a range of future scenarios. Hajat et al [1] derived heat and cold-mortality exposure–response functions for the UK, and used 2030s, 2050s, and 2080s climate and population growth and aging projections to estimate future temperature-attributable mortality. A similar method was used to estimate future heat and cold mortality in the UK and Australia [11]. Vicedo-Cabrera et al [20] estimated how heat mortality may be affected in a number of locations around the world, given a range of climate scenarios, but with fixed populations. Studies that investigate temperature-mortality relationships typically use a ‘hockey stick’ regression function, where the risk of mortality increases above or below a certain temperature threshold. These thresholds can differ across climate regions, with individuals in hotter climates better adapted to heat. Populations have shown the potential to adapt to hotter weather conditions over time [21], although a recent study found no evidence of decreasing susceptibility to heat and cold in London [22].

Given the potential for housing to modify temperature exposures, a number of studies have combined building physics and health models to project temperature-related health impacts due to housing. Taylor et al [23] used building simulation to model indoor temperature exposures for individual dwellings, and then combined these with modelled Urban Heat Island (UHI) temperatures and local populations by age group in order to estimate the spatial variation in heat mortality risk in London, UK. Liu et al [24] combined simulated indoor temperatures with local projected future temperatures for Sheffield, UK, in order to predict the spatial variation of heat mortality under different climate scenarios. Further studies have examined how modifications to housing may alter temperature mortality risks. Taylor et al [25], [26] investigated how heat-related mortality in the West Midlands, UK, might change, given energy efficiency improvements and the installation of external shutters under current and future climates. This study, however, did not include cold effects, nor a changing population and subsequent changes to housing to accommodate population growth. Hamilton et al [27] estimated how building adaptations may reduce cold-related mortality under the current climate only, and without population changes and growing housing stock. We are not aware of any study to-date that seeks to estimate the heat and energy impacts of the combined effects of climate change, population aging, and housing adaptation scenarios.

This study thus seeks to examine how changes to the climate, population, and housing stock will change future (2030s and 2050s) heat and cold mortality and space heating energy use in London, UK. In particular, we aim to answer the following: 1) given expected changes in temperature from climate change, and an ageing population, how much of a role does housing play in protecting against temperature-related health effects during a typical year; and 2) what are the relative contributions of climate, population, and housing factors on changes to such mortality and energy use?

## 2 Methods

**Fig. 1:**
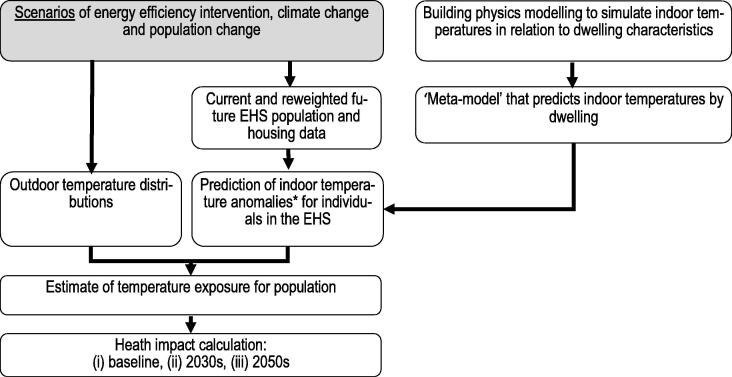
Schema of the modelling methods used for computation of heat and cold mortality. * ‘Anomaly’ refers to the difference between the temperature parameter for the specific dwelling and the average for the housing stock as a whole.

The methods used to model temperature-related mortality are summarized in Fig. 1.

### 2.1 Future population and housing projections

The 2010–2011 English Housing Survey (EHS) [28] is a representative survey of around 16,000 dwellings and their occupants across England. The EHS contains weighting values to enable the surveyed households to be projected to the regional and national English stocks and populations. To account for future scenarios with changes to the population in different age groups and housing stock, we re-weight this baseline EHS for London to represent the population and stock in 2030 and 2050. The re-weighting method is described in detail in Appendix 1.

**Fig. 2:**
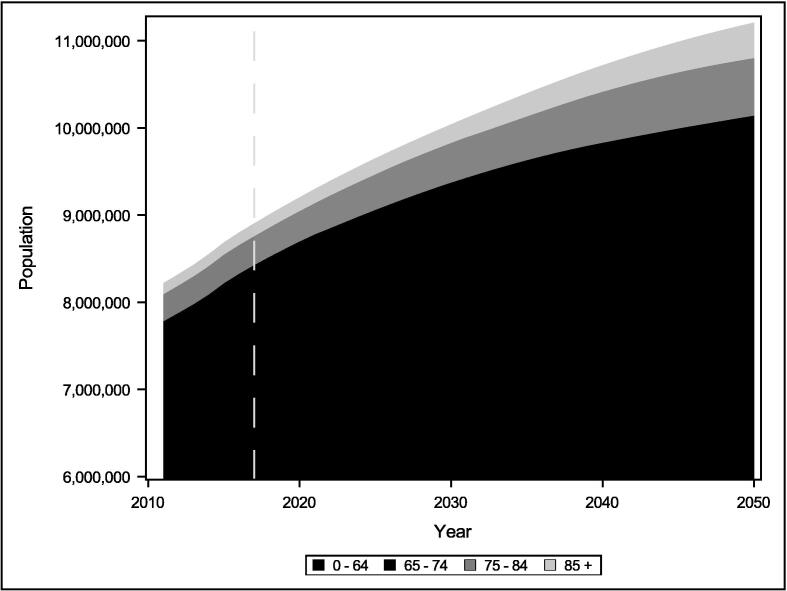
GLA population changes by age bracket for London. The dotted line shows the date from which projections begin.

Changes to the population and age profiles from 2010 to 2050 are based on the Greater London Authority (GLA) 2016-based housing-led population projections [29] (Fig. 2). We model four different scenarios under various climate change scenarios:

(i) The current population and housing stock;
(ii) The projected growth and aging of the population without any housing changes;
(iii) Population growth and aging with housing being retrofit and demolished at the current rate;
(iv) Population growth and aging with current rates of housing replacement, and housing retrofit at an ambitious rate.

In addition, we modelled changes with and without additional adaptations to protect against heat risks – namely, use of external shutters between 9 am and 6 pm for the period 1 May to 31 Aug.

Retrofit was assumed to occur only in EHS dwellings which are not currently insulated to the minimum thermal conductivity (U-value) possible according to the dwelling age and fabric type [30]. *Deep retrofit* includes insulation of walls (cavity wall insulation or internal solid wall insulation), floors, and roofs, and the installation of triple glazed windows; *partial retrofit* includes installation of loft and/or cavity or solid wall insulation. Post-retrofit changes to dwelling fabric U-values and permeability are described in more detail in Taylor et al [25]. The model is unable to account for changes in housing heating system efficiency and so estimates are for fabric-related interventions, and their associated reduction in dwelling air permeability, only. Current wall and loft retrofit rates for London were estimated from Department for Business, Energy and Industrial Strategy (BEIS) data on retrofits from 2013 to 2018 in London (Appendix 2) while ambitious rates are based on the need for 94% of the stock to have a deep retrofit by 2050 in order to meet targets set out in the 2008 Climate Change Act [31] (Table 1).

**Table 1:** Modelled changes to population and the existing housing stock from 2011 for various scenarios.

Scenario		(i) No Change		(ii) Population Only		(iii) Current Retrofit Rate		(iv) Ambitious Retrofit Rate	
Climate Epoch		2030s	2050s	2030s	2050s	2030s	2050s	2030s	2050s
Population Growth		None		Projected		Projected		Projected	
Housing Stock Change (%)	Demolition	–		–		2%	4%	2%	4%
	Deep Retrofit	–		–		–	–	33%	94%
	Partial Retrofit								
	Cavity Wall Insulation*	–		–		22.6%	46.5%	–	–
	Solid Wall Insulation*	–		–		1.2%	2.5%	–	–
	*Loft Insulation*	–		–		4.3%	7.8%	–	–
	Cavity & Loft Insulation*	–		–		1.1%	4.5%	–	–
	Solid Wall & Loft Insulation*	–		–		0.06%	0.25%	–	–

* Of cavity or solid-walled stock.

We assumed demolition of housing would continue at the 2015/2016 rate of 3100 a year for houses built between 1918 and 1970. New dwellings, constructed to current building regulations, replace demolished dwellings as well as housing the additional new population.

### 2.2 Indoor temperature modelling

The development and assumptions of the indoor temperature model are described in detail in various publications [23], [25], [26], [32]. To summarise, a building physics model, EnergyPlus [33], was used to simulate indoor temperatures for dwelling archetypes representative of the London stock. This modelling uses empirical data from national surveys to inform dwelling characteristics such as floor area, ceiling height, building fabric energy efficiency, wall type, window size, orientation, and airtightness. Modelled occupant behaviours included internal gains from appliances, while occupants were assumed to open windows 1/3 of their openable extent from May – September when indoor temperatures exceeded 22°C. Modelled indoor temperature results have been compared against large datasets of monitored temperatures, indicating that the model captures differences in dwelling overheating risk [34]. The modelled distribution of ventilation rates are also comparable to monitored rates in the English housing stock [35].

The results of this time-intensive modelling were then used to develop a more efficient ‘meta-model’ that predicts key indoor temperature parameters for a given dwelling using a similar range of dwelling characteristics. The development of the building stock *meta*-model has been described in detail in Symonds et al [32]. Briefly, a large number of EnergyPlus models were run in parallel using High Performance Computing (HPC). Model inputs and outputs were used to train and validate a set of Artificial Neural Networks, capable of rapidly estimating indoor conditions and space heating energy consumption using a limited set of dwelling characteristics as input. The metamodel has previously been used to estimate overheating and space heating energy consumption in the West Midlands [25], [26], and here we use it to predict key parameters for all individuals in the re-weighted variants of the EHS based on the dwellings they inhabit.

The predicted dwelling parameters we derived were ones needed as inputs to the epidemiological and energy use modelling, namely:

(i) The daily maximum indoor temperature, $T\left(in\right)$;
(ii) The ‘Standardized Indoor Temperature’ (SIT), which is the predicted winter indoor temperature at mid-afternoon on a day when the day maximum outdoor temperature was 5°C [14]; and
(iii) Household annual space heating energy consumption (kJ).

From these we computed temperature anomalies, which we define as the difference between the temperature exposure for an individual in the EHS inside their dwelling and the population average indoor temperature exposure using the approach used by Taylor et al [23], [25] for heat. Thus, the heat anomaly for individual *i* on day *k*, $\Delta T{\left(heat\right)}_{i,k}$ was defined as the difference between the indoor temperature in that individual’s house, $T{\left(in\right)}_{i,k}$, and its average across all individuals in the population:

(1) $$\Delta T{\left(heat\right)}_{i,k}=\left(T{\left(in\right)}_{i,k}-\overset{-}{T{\left(in\right)}_{k}}\right)$$

Similarly, the cold anomaly for individual *i*, $\Delta {\mathrm{SIT}}_{i}$, was defined as the difference between the SIT for that individual’s dwelling and the average across the population:

(2) $$\Delta {\mathrm{SIT}}_{i}={\mathrm{SIT}}_{i}-\overset{-}{\mathrm{SIT}}$$

### 2.3 Heat and cold mortality

The indoor temperature parameters from 2.2 were then combined with outdoor temperature distribution and population data to compute the population exposure to heat and cold and the associated mortality impacts using a method adapted from Taylor et al [25] which is described here. All calculations were performed in SAS [36].

Baseline hourly weather data came from London Heathrow Airport for 2005 – 2014 [37], which includes a heatwave from Jun 26th – 30th, 2006. For future periods (2030s, 2050s), we used a Test Reference Year (TRY) weather file – also with hourly temperature data representing ‘typical’ monthly conditions for Heathrow under both medium and high CO_2_ emissions scenarios for several probabilistic projections (10%, 50%, and 90% percentiles) [38]. In addition, we use Design Summer Year (DSY) climate files for the same future scenarios to represent hot (but not extreme) summers. Detailed information on the future weather file development can be found in Eames et al [38]; briefly, these weather data were created by morphing baseline (1960–1990) weather data into future weather files using a Weather Generator for UKCP09 climate projections. We acknowledge two issues. Firstly, we use a more recent baseline period for our model than was used to generate the future climate data in order to make our baseline concurrent with the EHS and mortality data. Secondly, UKCP09 has recently been replaced by UKCP18, however we are not aware of any hourly weather files generated using the more recent projections.

The key assumption in this modelling is that personal exposure to heat and cold is defined by the combination of outdoor temperature and the temperature anomaly for heat or cold as defined above. Thus, for heat, we added the heat anomaly, $\Delta T{\left(heat\right)}_{i,k}$, to the outdoor temperature on the same day, $T{\left(mean,out\right)}_{k}$, to give the *effective* temperature exposure for heat for an individual in the population:

[3] $${T\left(mean,heateffective\right)}_{i,k}=T{\left(mean,out\right)}_{k}+\Delta T{\left(heat\right)}_{i,k}$$

And for cold, we added the cold anomaly, $\Delta {\mathrm{SIT}}_{i}$, to the outdoor temperature on the same day, $T{\left(mean,out\right)}_{k}$, to give the *effective* temperature exposure for cold for an individual:

[4] $${T\left(mean,coldeffective\right)}_{i,k}=T{\left(mean,out\right)}_{k}+\Delta {\mathrm{SIT}}_{i}$$

The assumption here is therefore that the dwelling modifies the outdoor temperature by an amount equal to the heat or cold anomaly. The rationale is as follows.

First, it is important to recognise that mortality risks are very well established in relation to *outdoor* temperature, but there is almost no direct evidence in relation to indoor temperatures, although there is evidence that housing modifies temperature-related mortality risk [14], [19]. But, at a given outdoor temperature, individuals in different dwellings will be exposed to a lower or higher than population average temperature by (on average) an amount equal to the dwelling temperature anomaly. The assumption we make is that this anomaly can be used to define the risk of heat- or cold-related mortality by moving an individual up or down the *outdoor* temperature-mortality function by an equivalent amount (giving the ‘effective temperature’). This is not to assume that the actual personal temperature exposure is the same as the effective temperature, but rather that the effective temperature reflects the distribution of risk for a given outdoor temperature defined by variations in indoor environments, with an average difference of risk across dwellings of zero.

To compute the mortality risks we used the ‘hockey-stick’ models published by Vardoulakis *et al* [11] in which the heat risk was defined as a (log-linear) increase in mortality above a threshold temperature for heat, $T_{h}$ (based on temperatures at lag 0 and 1 days) and that for cold was defined as a (log-linear) increase in risk below a cold temperature threshold, $T_{c}$ (based on temperatures at lags 0 to 27 days). The relative risks for heat (${\mathrm{RR}}_{h}$) and cold (${\mathrm{RR}}_{c}$) for different age groups are shown in Table 2.

**Table 2:** Relative risks for heat and cold, obtained from Vardoulakis et al [11].

Age (*a*)	Heat		Cold	
	Heat Threshold °C ($T_{h}$)	Heat RR per degree °C, (${\mathrm{RR}}_{h}$)	Cold Threshold °C ($T_{c}$)	Cold RR per degree °C, (${\mathrm{RR}}_{c}$)
0–64	19.6	1.028	13.3	1.012
65–74		1.025		1.023
75–84		1.048		1.025
85 -		1.064		1.035

Thus, the excess risk for a given outdoor maximum temperature above $T_{h}$ (based on the two day mean of lag 0–1 days) is given by:

(5) $$RelativeExcessRisk\left(heat\right)=\left[{\left({\mathrm{RR}}_{h}\right)}^{{T(mean,heateffective)}_{i,k\left[lag0-1\right]}-T_{h}}-1\right]$$

and zero when the temperature is below $T_{h}$.

Similarly, the excess risk for a given outdoor maximum temperature below $T_{c}$ (based on the mean of lag 0–27 days) is given by:

(6) $$RelativeExcessRisk\left(cold\right)=\left[{\left({\mathrm{RR}}_{c}\right)}^{{T(mean,coldeffective)}_{i,k\left[lag0-27\right]}-T_{c}}-1\right]$$

and zero otherwise.

These excess risks were used in conjunction with the number of deaths per each day of the year in London (averaged from 2005 to 2014) for the different age groups [39] to derive the totals of heat and cold attributable deaths under each of the modelling scenarios. This calculation method assumes no population adaptation to heat. To test how adaptation may potentially change heat attributable mortality in the future, we also calculated results assuming that $T_{h}$ shifts as the climate warms. Here, future $T_{h}$ was recalculated as the 93rd percentile temperature for typical years under the different climate scenarios.

### 2.4 Energy consumption estimates

We include a rough initial estimate of the change in space heating energy use due to the fabric retrofits and outdoor temperatures under the various climate and retrofit scenarios. The metamodel is capable of estimating a dwellings annual space heating requirement, based on a thermostatic set temperature of 21°C, on for 9 h on weekdays (7am – 9am and 4 pm – 11 pm) and 16 h (7am – 11 pm) on weekends from September – May, as informed by SAP [30]. It accounts for built form, fabric, solar and internal gains, and ventilation characteristics under current climate conditions, but does not account for the efficiency of the heating system. Therefore, we only estimate the theoretical change from existing energy consumption for dwellings relative to the current stock under different retrofit scenarios. We acknowledge that there is significant uncertainty in individual-dwelling energy consumption estimates but believe these are minimised when aggregated at stock-level for London.

We then apply a fractional adjustment to the stock-level space heating energy consumption for future climates. The weather-dependent demand for energy to heat a building can be quantified using the Heating Degree Hour (HDH) – or the number of degree-hours that the outdoor temperature drops below a threshold. The weather files are used to calculate HDHs for the different climate scenarios relative to a UK standard base temperature of 15.5°C [40] during heating hours, while modelled energy consumption is summed across the stock for dwellings. The fractional change in energy use is calculated as follows:

(7) $$F_{\mathrm{future}}=\left(\frac{E_{\mathrm{future}}}{E_{\mathrm{Base}}}\right)\times \left(\frac{{\mathrm{HDH}}_{\mathrm{future}}}{{\mathrm{HDH}}_{\mathrm{Base}}}\right)$$

where $E_{\mathrm{future}}$ is the stock energy consumption under the different future scenarios, $E_{\mathrm{Base}}$ is the 2010–2011 stock energy use, ${\mathrm{HDH}}_{\mathrm{future}}$ is the HDH under different climate scenarios, while ${\mathrm{HDH}}_{\mathrm{Base}}$ are the HDHs for the baseline climate. This simplified approach allows for an approximate estimate of stock-level space heating energy consumption that provides important additional context to the health calculations.

## 3 Results

Results for the outdoor and indoor temperature exposures and the resultant mortality and energy consumption are shown below.

### 3.1 Heat and cold exposures

**Fig. 3:**
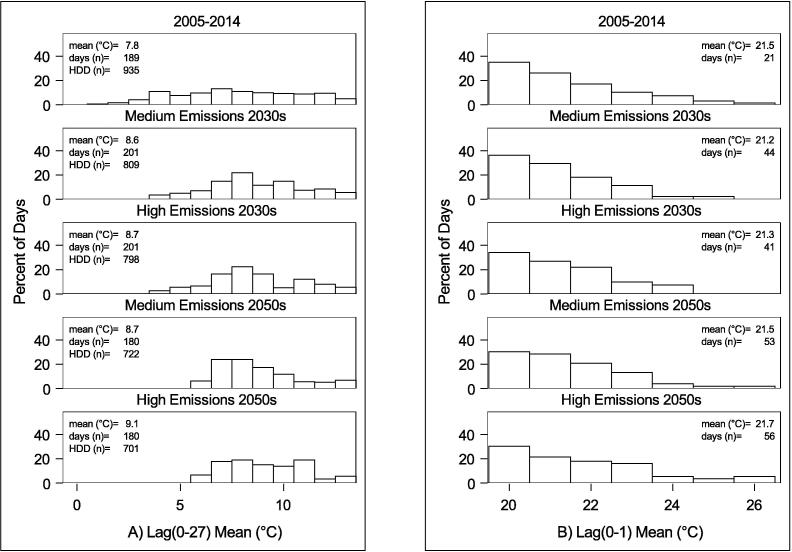
A) The distribution of mean Lag (0–27) temperatures when below the London threshold of 13.3°C; B) The distribution of Lag (0–1) Mean temperature when above the London threshold of 19.6°C. Both are for 50th climate probability and ‘typical’ years. The mean lag (0–27) and lag (0–1) when thresholds are exceeded, the number of days (n) when thresholds are exceeded, and the HDDs are shown in the insets.

The differences in outdoor temperatures for typical years under the climate scenarios can be seen in Fig. 3. Changes in cold weather are shown in Fig. 3A, with the distribution of days by Lag (0–27) temperatures on days below the cold mortality threshold in London. This indicates a reduction in the total number of ‘cold’ days; an increase in the mean Lag (0–27) temperatures on such days; a decrease in the number of very cold days, and a decrease in the HDDs required in the future. Fig. 3B shows an increase in ‘hot’ days, or days exceeding the heat mortality threshold, under future climate scenarios. It shows an increase in the number of days that exceed the heat mortality threshold, as well as an increase in the mean Lag (0–1) temperature on such days, and a greater number of very hot days.

**Fig. 4:**
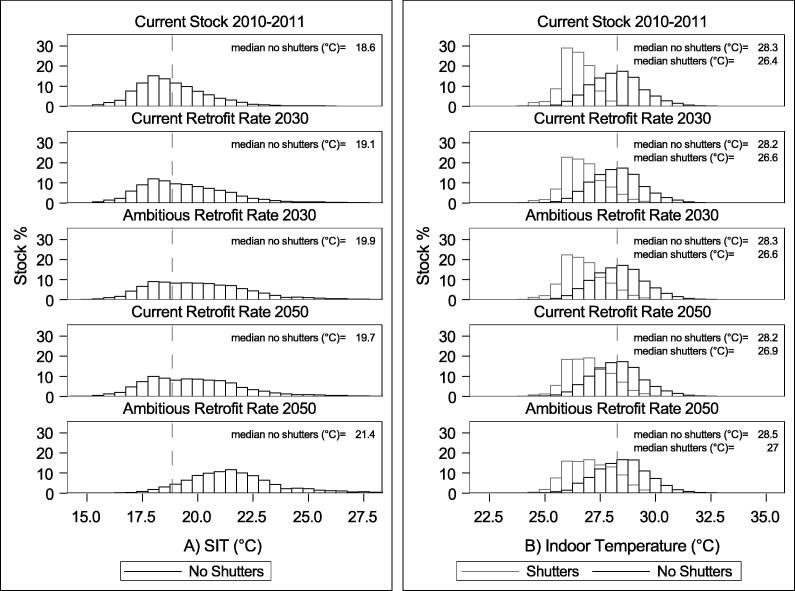
A) Distribution of SIT under no change, current retrofit rate, and ambitious retrofit rate; B) Distribution of summer indoor temperatures at a mean lag (0–1) maximum outdoor temperature of 24.8°C under no change, current retrofit rate, and ambitious retrofit rate with/without shutters. The dotted reference line indicates the mean of the current stock.

The changes to the temperature distribution of the housing stock under the different housing adaptation scenarios can be seen in Fig. 4. Fig. 4A shows the distributions of SIT for the modelled London stock under current and future adaptation scenarios. The current retrofit rate will lead to a 0.5°C increase in the median SIT by the 2030s and 1.1°C by the 2050s, while the ambitious rate will increase median SIT by 1.3°C by the 2030s and 2.8°C by the 2050s. Retrofits show insignificant changes in stock median indoor temperatures (${T\left(mean,heateffective\right)}_{i,k}$) during hot weather (Fig. 4B), with the exception of the ambitious rate which is predicted to increase the median by 0.2°C by the 2050s. During hot weather, shutters are able to substantially reduce median indoor temperatures by 1.3°C to 1.9°C relative to the non-shuttered stock across retrofit scenarios.

### 3.2 Heat and cold mortality

**Fig. 5:**
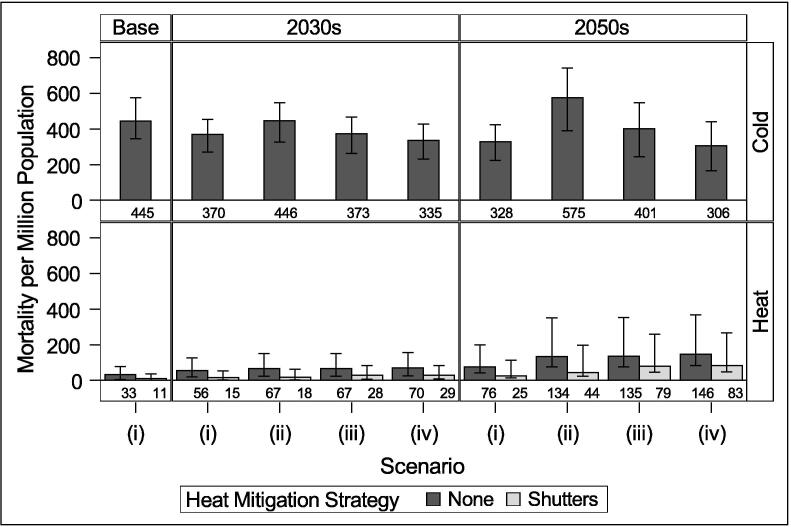
Cold and heat mortality per million population under medium emissions scenario and different housing and population scenarios: (i) No population changes or retrofit; (ii) Population changes only; (iii) Population changes and current retrofit rates; (iv) Population changes and ambitious retrofit rates. Error bars indicate 10% and 90% climate probabilities.

**Fig. 6:**
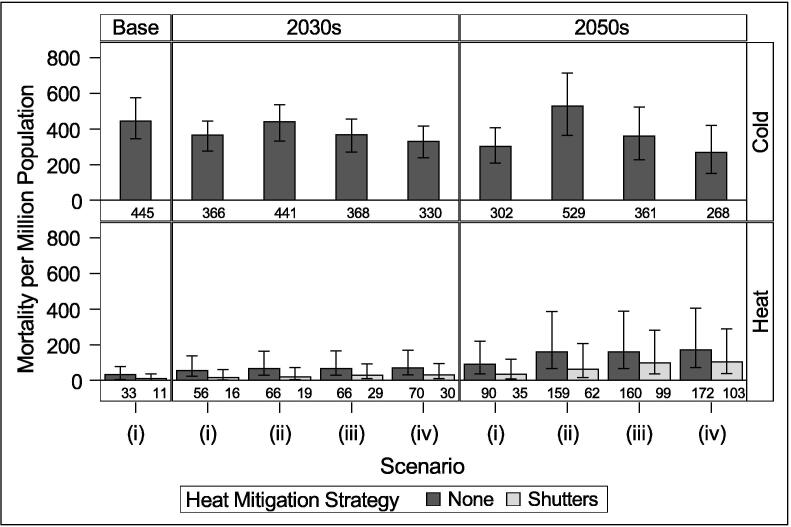
Cold and heat mortality per million population under high emissions scenario and different scenarios: (i) No population changes or retrofit; (ii) Population changes only; (iii) Population changes and current retrofit rates; (iv) Population changes and ambitious retrofit rates. Error bars indicate 10% and 90% climate probabilities.

Projected absolute heat and cold-related mortality for the different scenarios are shown in Fig. 5 (medium emissions) and Fig. 6 (high emissions). We estimate an annual average of 445 cold-related deaths per million and 33 heat-related deaths per million in London from 2005 to 2014. The summer of 2006 – during which the heatwave occurred – had the highest estimated heat-attributable mortality of 77 per million over the duration of the summer.

Results indicate that cold will remain the greater temperature-related risk in the future for London for typical years. Without accounting for population or housing changes (Scenario i), there is a decrease in cold-related mortality and an increase in heat-related mortality due to climate effects relative to the baseline, as would be expected. When aging is accounted for (Scenario ii), there is a substantial increase in both heat and cold mortality by the 2050s due to the increased temperature vulnerability of the population. If energy efficiency retrofits continue at the current rate (Scenario iii), we would expect to avoid 73 annual deaths per million from cold in the 2030s and 168–174 per million by the 2050s, while annual heat-related deaths increase by around 1 death per million by the 2050s. Under an ambitious retrofit policy (scenario iv), around 111 cold deaths per million are avoided annually by the 2030s and 261–269 by the 2050s, while annual heat-related deaths increase by 3–4 by the 2030s and 12–13 by the 2050s.

**Fig. S3.1:**
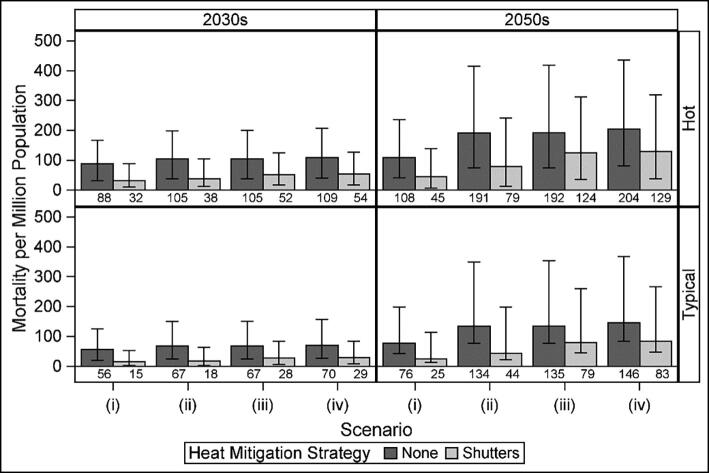
Heat-related mortality for future ‘typical’ and ‘hot’ summers under medium emissions and different housing and population scenarios: (i) No population changes or retrofit; (ii) Population changes only; (iii) Population changes and current retrofit rates; (iv) Population changes and ambitious retrofit rates. Error bars indicate 10% and 90% climate probabilities.

**Fig. S3.2:**
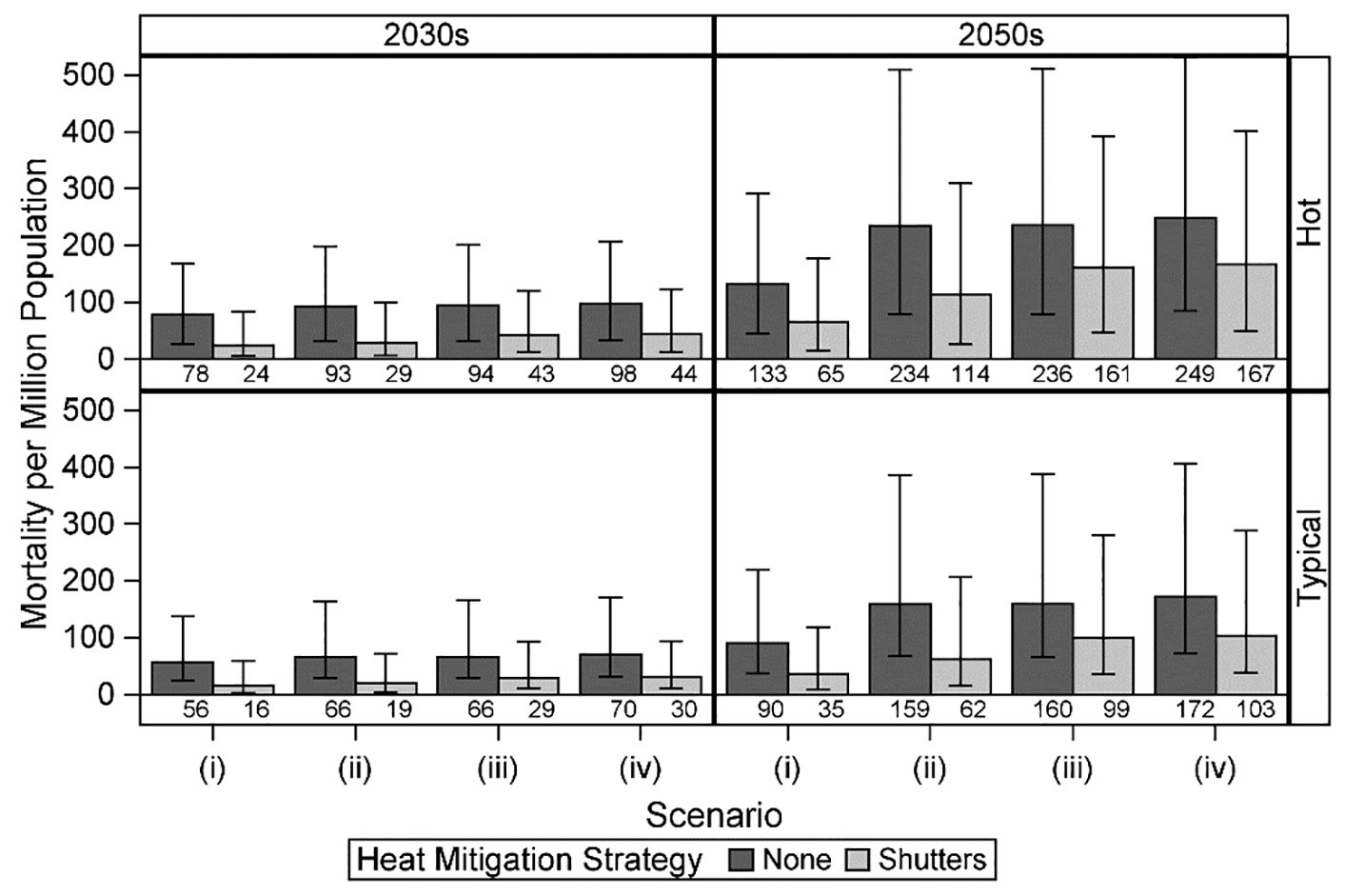
Heat-related mortality for future ‘typical’ and ‘hot’ summers under high emissions and different housing and population scenarios: (i) No population changes or retrofit; (ii) Population changes only; (iii) Population changes and current retrofit rates; (iv) Population changes and ambitious retrofit rates. Error bars indicate 10% and 90% climate probabilities.

**Fig. S3.3:**
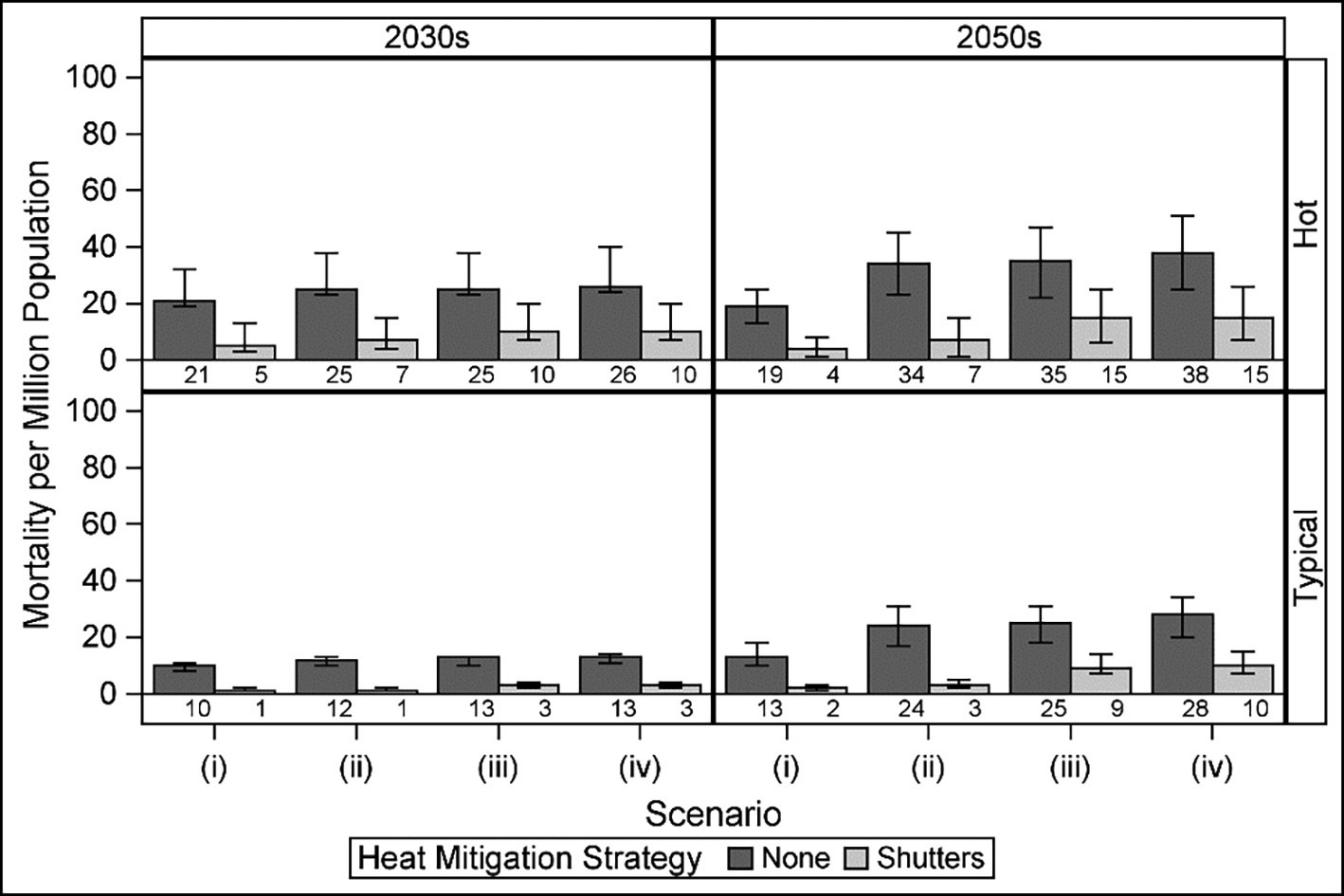
Heat-related mortality for future ‘typical’ and ‘hot’ summers under medium emissions and different housing and population scenarios, with population adaptation to heat: (i) No population changes or retrofit; (ii) Population changes only; (iii) Population changes and current retrofit rates; (iv) Population changes and ambitious retrofit rates. Error bars indicate 10% and 90% climate probabilities.

**Fig. S3.4:**
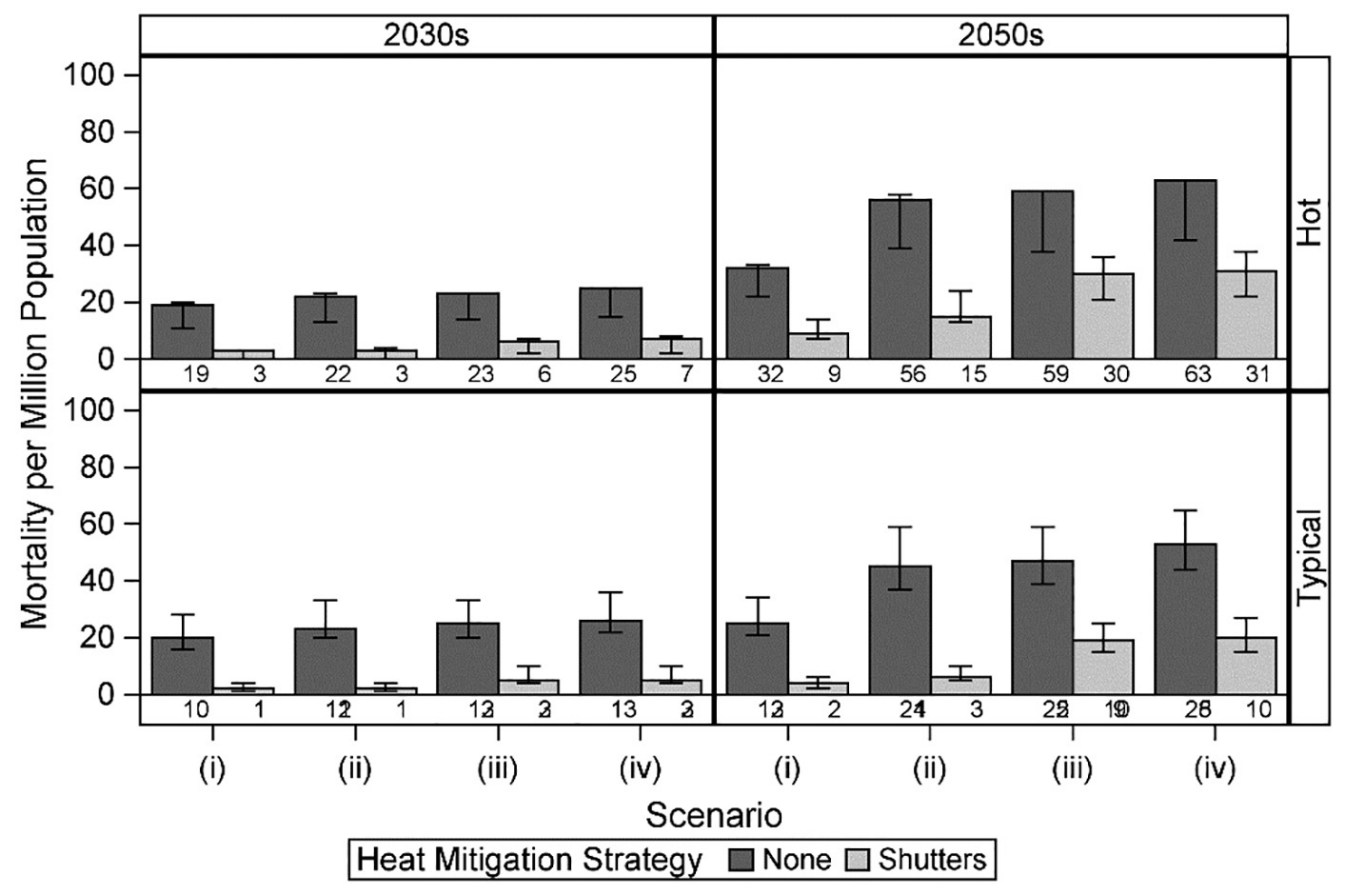
Heat-related mortality for future ‘typical’ and ‘hot’ summers under high emissions and different housing and population scenarios, with population adaptation to heat: (i) No population changes or retrofit; (ii) Population changes only; (iii) Population changes and current retrofit rates; (iv) Population changes and ambitious retrofit rates. Error bars indicate 10% and 90% climate probabilities.

The results for hotter future summers and with population adaptation can be seen in Appendix 3. Relative heat-attributable deaths increase under the hotter summers, but the relative changes in risk following retrofit are similar to those during typical summers. Under medium emissions, hot summers may see ambitious retrofit increase annual heat attributable mortality by around 4 people per million by the 2030s and 13 people per million by the 2050s relative to no retrofit (Fig. S3.1). Under high emissions, retrofit increases annual heat attributable mortality by 5 per million by the 2030s and 15 per million by the 2050s during hot summers (Fig. S3.2). Population adaptation is able to substantially reduce heat-attributable mortality during typical and hot summers to numbers similar to the baseline period average (Figs. S3.3 and S3.4).

Installing external shutters on dwellings is estimated to significantly reduce heat mortality risk. We estimate that shutters would have reduced mortality during the 2006 summer from 77 per million to 36 per million, or by 332 people. Without any retrofit (Scenario ii), shutters may lead to 47–49 deaths per million (72–73%) avoided by the 2030s and 90–97 deaths per million (61–67%) by the 2050s. When coupled with the current rate of retrofit (iii), shutters may reduce mortality by 56–58% by the 2030s and 38–41% by the 2050s; at the ambitious rate (iv), they reduce mortality by 57–59% by the 2030s and 40–43% by the 2050s. While the percentage of heat mortality avoided due to shutters decreases under future hotter climates, shutters are still highly effective at reducing risk (Figs. S3.1 and S3.2).

### 3.3 Energy for space heating

**Fig. 7:**
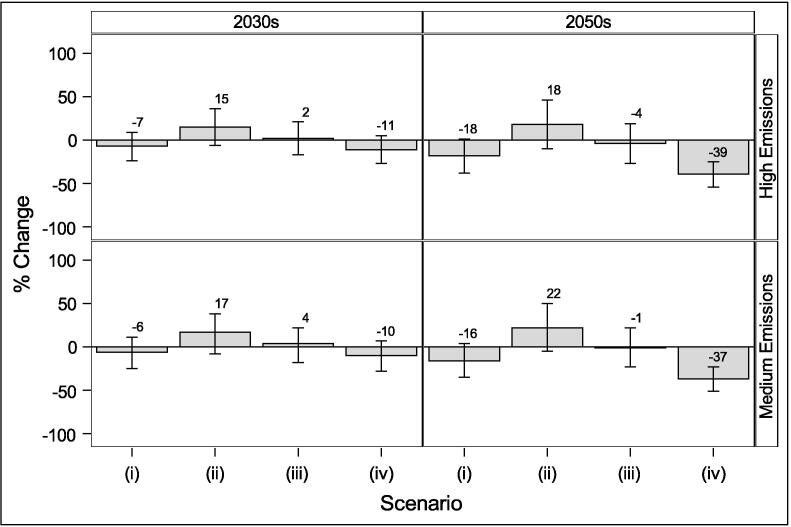
Energy consumption for space heating for different retrofit scenarios: (i) No population changes or retrofit; (ii) Population changes only; (iii) Population changes and current retrofit rates; (iv) Population changes and ambitious retrofit rates. Error bars indicate 10% and 90% climate estimates.

Estimated changes to stock level energy consumption for space heating from current levels due to climate change, population increases, and housing fabric retrofit scenarios can be seen in Fig. 7. Climate-only effects would lead to a 6%-7% reduction in energy consumption by the 2030s and 16–18% reduction by the 2050s depending on the emissions scenario. However, with the growing population, energy consumption is projected to increase by 15–17% by the 2030s and 18–22% by the 2050s. Retrofitting at the current rate can largely offset that increase, but more ambitious retrofit is necessary to lead to an overall reduction in the future. These estimates represent only the changes in energy required for heating the dwellings assuming a theoretical 100% efficient heating system. Further reductions would be possible with more efficient heating systems such as heat pumps.

## 4 Discussion

### 4.1 Summary of findings

Our results indicate that population aging significantly increases the population vulnerability to heat and cold-related mortality in future climates. Without any adaptation of the building stock, we project an increase in both heat and cold mortality between the baseline period and 2050s during typical years. While winters become warmer, cold mortality continues to increase due to the larger population of temperature-vulnerable elderly. Hotter summers and a more vulnerable population lead to a large relative increase in heat-related mortality. By the 2050s, without changes to housing or population adaptation, we predict a 4 to 5-fold increase in heat-related mortality and 1.2 to 1.3-fold increase in cold mortality relative to the 2005 – 2014 baseline.

**Fig. 8:**
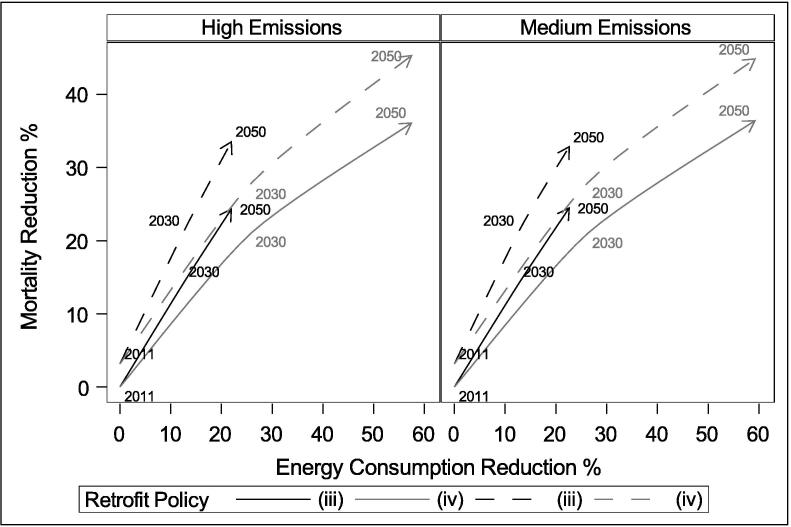
Projected potential future reductions in temperature-related mortality and space heating energy use for scenarios iii (current rate) and iv (ambitious rate), relative to the no-retrofit scenario (ii). Dashed lines are with shutters.

Energy efficiency and heat mitigation adaptations to the housing stock have the potential to modify cold and heat related mortality, whilst reducing energy consumption. The current retrofit rate may avoid 168–174 cold-related deaths per million (a decrease of around 30%) by the 2050s, increasing heat-related mortality by around 1 death per million (0.3 – 0.8% increase), and reducing energy consumption by 1–4%. A more ambitious retrofit rate is predicted to avoid 261–269 cold deaths per million (a decrease of around 50%) by the 2050s, whilst increasing heat mortality risk by around 12–13 deaths per million (or 8%) and decreasing energy consumption by 37–39%. The protective benefits of retrofit for cold exposure are greater than any disbenefits for heat exposure. Fig. 8 shows how the different policy scenarios may change temperature-related mortality and energy consumption in the future under different emission scenarios.

External shutters are predicted to effectively mitigate heat mortality. By the 2030s, shutters avoid 37–49 heat deaths per million population (56–73% reduction), depending on the climate and retrofit scenario. By the 2050s, shutters could avoid 56–97 heat deaths per million population (35–67%). While the percent reduction in heat mortality following shutters decreases slightly with increased energy efficiency, and more significantly under hotter climates, they are still predicted to be highly effective at avoiding heat mortality.

An increased rate of retrofit is critical to reduce space heating energy use. In the absence of changes to heating systems, more ambitious levels of retrofit are necessary in order to reduce the energy demand of the stock, and a warming climate cannot be relied on to reduce space heating consumption by itself given a growing population. While increased energy efficiency may marginally increase the risk of heat mortality, this increase is predicted to be relatively easily offset in London using external shutters. It is therefore important that houses undergoing retrofit include adaptations for heat mitigation.

### 4.2 Comparison with previous studies

Our estimates of changes in heat and cold related mortality due to climate change and without changes to housing factors are compared with previous estimates in Table 3. Vardoulakis et al. [11] and Hajat et al. [1] both estimated that for a fixed population in the UK, rising temperatures would lead to increased heat mortality and decreased cold mortality in future decades. However, when taking into account the predicted growth and aging of the UK population, the beneficial effect of warmer winters was diminished and the negative impact of higher summer temperatures was exacerbated when applied to a larger and more vulnerable UK population. Our method predicts comparably fewer heat-related deaths under current conditions and more under future conditions for cold.

**Table 3:** Comparison of results (heat and cold deaths per year per million population) with previous studies for London.

		This study	Vardoulakis et al. (2015)[11]	Hajat et al. (2014) [1]
Heat	Baseline	33	47	40
	2050s Medium	134	78	113 (40–210)
	2050s High	159	91	–
Cold	Baseline	445	605	773
	2050s Medium	575	515	584 (532–675)
	2050s High	529	490	–

The primary drivers of the increase in mortality in this model are the population projections, which indicate a 41% increase in the over 85 population by 2030 and 176% increase by 2050 relative to the 2011 population. Similarly, the age group 75–84 is predicted to increase 47% by 2030 and 120% by 2050. For cold, the relatively modest decrease in cold exposure days (6% decrease by 2030 and 16% by 2050) and mean lag (0–27) temperature on these days does not offset the increased vulnerability of the population. For heat related mortality, the increased population vulnerability is exacerbated by increased frequency and mean lag (0–1) temperatures on hot days. The use of different underlying climate data also contributes to the different results between studies.

### 4.3 Strengths and limitations

The modelling framework has been applied here for temperature-related mortality and energy use, but is flexible. It can be extended in the future to look at different indoor environmental exposures and health outcomes (for example indoor air pollution), housing change or population growth rates, other regions in England, and different climate scenarios. It can be extended to 2080, however we have not done this here due to the compounded uncertainty in population and housing retrofit projections.

We use openly available climate data in this model. To be consistent with the time period of the housing and mortality data, we used baseline climate data from 2005 to 2014, rather than the baseline data that was used to generate the future weather files (1961–1990). Hourly weather data was used to run the building physics models and calculate HDDs, but future versions of the model could use daily averages from, for example, UKCP18 [41]. Weather data representative of ‘typical’ years excludes extreme weather events such as heatwaves - which are projected to become more frequent in the future. We used typical years in the analysis in order to compare between years, emission scenarios, and trade-offs between heat and cold risks during normal years, although results for hotter summers are shown in the Appendix. In London, the largest burden of heat deaths are thought to occur during isolated hot days or more frequent moderately hot days, with heat waves accounting for fewer than half of all heat-related deaths [9].

#### 4.3.1 Housing model

Reweighting assumes that people continue to inhabit the housing types that they currently prefer, and that new housing reflects the current choices of the different age groups. We have not, however, accounted for changes in housing preferences – for example, densification could lead to smaller housing and increased demand for housing could lead to the conversion of existing houses into converted flats. We have not accounted for changes in building trends, such as the current trend towards increased glazing, which may increase heat risk further. In urban areas, outdoor temperatures may vary due to the UHI; we have not accounted for this spatial variation in both heat and cold exposures as the EHS does not include spatial data. Population growth may lead to increased urban density, which could increase UHI temperatures.

We have assumed that new buildings are built under the current – relatively unambitious – UK building standards requirements for energy efficiency. We used a baseline from Jan 2013 to Dec 2018 to estimate the ‘current’ retrofit rate, however, in the more recent years there has been a 95% drop in insulation rates. If this continues, future estimates are likely to more closely resemble the scenario with population growth but no retrofit. Policies aimed at reducing fuel poverty using fuel subsidies may also help reduce excess winter mortality, but have an energy cost, making retrofit a preferable long-term strategy. In solid walled buildings, we assume that insulation is installed internally; this can increase the risk of summertime overheating [42], so our predictions of heat-related mortality in retrofitted dwellings may represent an overestimate where external insulation is possible.

For hot weather, we have not modelled the effects of air conditioning, despite indications it is an important protective factor for heat mortality in the U.S. [43]. This is because of the low prevalence of air conditioning units in English housing - estimated at 3% [44] - and because passive systems such as shutters are preferable to active systems due to the need to reduce energy consumption. However, in the future heat pumps may be more widely available for both heating and cooling. The model presented in the body of the manuscript accounts for some adaptive occupant behaviour due to the assumed window opening during hot weather, but no other adaptation behaviours were assumed such as changes to clothing levels, as we have focused here on adaptations to dwellings only. Results assuming complete adaptation are shown in Appendix 3, and predict no increase in heat-related mortality relative to the baseline period.

The energy consumption estimates are a simplified initial estimate based on climate change, the growth of the housing stock and retrofit of the building envelope only, and the changes should be considered indicative only. For example, the energy calculations do not account for the rebound effect, where households that upgrade their energy efficiency do not save energy because they maintain their houses at a warmer temperature. Increased efficiency of heating systems is not modelled but will be an important means of reducing energy consumption in buildings.

#### 4.3.2 Health impact calculations

We model adaptation of the housing stock assuming current temperature-mortality relationships - which implies no population adaptation or acclimatisation to heat as the climate warms via behavioral, physiological, or technological means. We also test the model outputs assuming complete adaptation, which results in future mortality levels remaining similar to current levels. There is mixed evidence to suggest population adaptation to heat in London [21], [22], [45], and our results here are intended to show the range of possible outcomes.

Gasparrini et al. [46] modelled the effects of climate change on heat and cold mortality for a fixed population, and estimated that changes in future temperature alone would lead to reductions in cold mortality and increases in heat mortality; Hajat et al. [1] and Vardoulakis et al. [11] also showed similar results for fixed populations, but that reductions in cold mortality were diminished when considering future changes to the UK population. Our results similarly suggest that temperature changes associated with climate warming would lead to decreased winter mortality if no other changes to the population are assumed.

We assumed that heat and cold exposure occurs in the home. While we acknowledge that many will be away from home during the day, mortality is dominated by deaths amongst the most vulnerable who are more likely to remain at home. For heat, indoor temperature modification of mortality is calculated using a relationship between excess mortality and outdoor temperatures; in the absence of a specific indoor temperature-mortality relationship, this seems a reasonable proxy. In contrast, the SIT-mortality relationship is derived using information on indoor temperatures and is assumed to modify the cold-mortality relationship equally across age groups.

While we have focused on mortality due to temperature exposures, there are important additional potential health implications of energy efficiency retrofits that we have not considered here. Increased energy efficiency may lead to an increase in indoor air pollution if additional purpose-provided ventilation is not installed. This may lead to an appreciable increase in risk. For example, radon levels have been found to increase following retrofit [47], while increased energy efficiency has been associated with increased asthma, cardiovascular disease (CVD), and chronic obstructive pulmonary disease admissions [48], [49]. Conversely, increased energy efficiency can have a substantial positive impact on mental health [14]. Previous health impact assessments have estimated an increase in population quality adjusted life years if retrofits are installed with appropriate additional ventilation and a net decrease if installed without [27].

#### 4.3.3 Implications for policy

A more ambitious housing retrofit policy is required to meet energy efficiency targets. We project that the potential reduction in cold-related mortality from retrofit is greater than the increase in heat-related mortality, and that there is potential to offset increases in heat mortality using simple and low-cost passive heat mitigation strategies. Energy efficient retrofit regulations in the UK require provisions for ensuring that building ventilation is not compromised; such regulations should also require parallel installation of heat mitigation adaptations in order to offset any increased risk during summer whilst providing benefits for winter health and comfort and energy consumption.

Whilst the annual mortality from heat exposure in the future is predicted to remain below that from cold in the UK, the heat burden will increase substantially due to the warming climate and aging population. Additional policies aimed at supporting the most vulnerable, such as public ‘cool’ spaces, will also be critical to reduce heat risk. While evidence indicates WFPs may help reduce mortality, they treat the symptoms rather than underlying cause of cold homes and do nothing to reduce energy consumption.

## 5 Conclusions

We have described the modelling of housing modification of temperature-related mortality, applying a building physics based metamodel to a representative housing stock dynamically weighted to account for changes to the population and housing over time. The described modelling framework is flexible, and can look at various climate scenarios, retrofit rates, and regions of England. In the future, it can be extended to include additional indoor health hazards.

We predict increases in temperature-attributable mortality by the 2030s and 2050s, driven by an aging population, and with heat-related mortality having the largest relative increase due to the higher temperatures. Housing can help reduce overall temperature-related mortality. At the current rate, during typical years retrofits are predicted to avoid 73 annual cold-related deaths per million by the 2030s and 168–174 per million by the 2050s; more ambitious retrofit rates could see this increase to 111 and 261–269, respectively. There is a comparatively smaller increase in annual heat-related mortality from retrofits of around 1 per million by the 2030s and 2050s under current retrofit rates, and 12–13 by the 2050s under more ambitious rates.

Risks from heat can be significantly reduced using external shutters. Our modelling indicates that they can avoid 38–73% of heat-attributable mortality during typical summers, depending on the climate and retrofit scenario. Their effectiveness at reducing indoor temperatures means that they are capable of more than offsetting any increase due to retrofit, and they – or similarly effective adaptations - should be implemented during dwelling retrofit in London. An ambitious retrofit rate is critical to decrease the space heating energy consumption of the housing stock, therefore including a means of mitigating this potential increase in indoor temperatures in energy efficient housing will be required.

## Declaration of Competing Interest

The authors declare that they have no known competing financial interests or personal relationships that could have appeared to influence the work reported in this paper.

## Appendix 1

The EHS was re-weighted to incorporate projected changes to the population and housing stock by 2030 and 2050. The process is as follows:

1. EHS occupant data form the basis for the stock, where each row is an individual in the population. EHS housing data is joined to the population data; dwellings occur more than once when there are multiple occupants in the dwelling. The EHS weights for individuals are calibrated to the 2011 ONS population for different age groups.

2. Population changes from 2010 to 2011 to future periods are applied by multiplying the fractional change in population for different age groups (0–64, 65–74, 75–84, 85-) to the current weightings for all London individuals in the EHS based on their age band:

(1) $${\mathrm{Wf}}_{a}={\mathrm{Wc}}_{a}\times R_{a}$$

where ${\mathrm{Wf}}_{a}$ is the future weight of an individual of age class *a;* ${\mathrm{Wc}}_{a}$ is the 2010–2011 (baseline or current) weight of an individual of age class *a*, and $R_{a}$ is the fractional change for an individual of age class *a* between the current (2010–2011) period and year *y*. For example, 2030 is:

(2) $$R_{a}=\frac{{\mathrm{Population}(2030)}_{a}}{{\mathrm{Population}(2011)}_{a}}=\frac{\sum {\mathrm{Wf}(2030)}_{a}}{\sum {\mathrm{Wc}}_{a}}$$

The numerator is the population size of age class *a* in year 2030 and the denominator is the counterpart current population size.

3. Each individual lives in a dwelling type (*d*). In the future, the housing stock will undergo changes. New houses will need to be constructed according to building regulations to accommodate the growing population; some existing houses will be demolished; and some of the stock will be retrofit to higher energy efficiency standards, either partially or completely. To account for this, each EHS entry was subdivided into four entries:

3a. Occupants in original dwellings (*d = original*), where buildings remain the same as in the 2010–2011 EHS.

3b. Occupants in new dwellings (*d = new*), where building geometry remains the same, but fabric energy efficiency and airtightness parameters are updated to meet current building regulations.

3c. Occupants in deep retrofit dwellings (*d = deep retrofit*), where geometry remains the same but building fabric energy efficiency and airtightness parameters are updated as described above to meet deep retrofit requirements.

3d. Occupants in partially retrofit dwellings (*d = partial retrofit*), where geometry remains the same but building fabric energy efficiency and airtightness parameters are updated as described above for partial retrofits.

4. Reweighting is then applied to the subset stock as follows:

4a. The population living in deep and partially retrofit dwellings are weighted according to the rate of retrofit:

(3) $${\mathrm{Wf}}_{a,d=deepretrofit}={\mathrm{Wc}}_{a,d}\times R_{\mathrm{deepretrofit}}$$

(4) $${\mathrm{Wf}}_{a,d=partialretrofit}={\mathrm{Wc}}_{a,d}\times R_{\mathrm{partialretrofit}}$$

Where ${\mathrm{Wc}}_{a,d}$ is the original population in age class *a* living in dwelling type *d*, and $R_{\mathrm{deepretrofit}}$ and $R_{\mathrm{partialretrofit}}$ are the deep and partial retrofit rates, respectively.

b. The population living in original dwellings is reweighted according to the removal of the original stock via a rate of demolition ($R_{\mathrm{demolition}}$), and the conversion of dwelling via retrofit from above using a population balance argument:

(5) $${\mathrm{Wf}}_{a,d=original}={\mathrm{Wc}}_{a,d}\times \left(\left({1-R}_{\mathrm{demolition}}\right)-R_{\mathrm{deepretrofit}}-R_{\mathrm{partialretrofit}}\right)$$

c. Individuals living in new dwellings are weighted according to the need to accommodate the growing population and replace the older stock that is removed via demolition:

(6) $$W_{f,a,d=new}=W_{c,a,d}\times \left(R_{a}-1+R_{\mathrm{demolition}}\right)$$

## Appendix 2

Partial retrofit rates for London are derived from the Household Energy Efficiency Statistics Detailed Report tables for 2019 (BEIS, 2019). These indicate that 80,067 cavity wall insulations, 6863 solid wall insulations and 43,063 loft insulations occurred in London via the Energy Company Obligation, from Jan 2013 to Dec 2018, within a stock containing 3,447,294 households in London with at least 1 resident. The 2010–2011 EHS indicates that 39% of dwellings in London have cavity walls

We estimated the annual probability of cavity wall and loft insulation retrofit of cavity walled dwellings as:

$$P\left(Cavity\&Loft\right)=P\left(Cavity\right)xP\left(Loft\right)=\left(\frac{\frac{80,067}{5years}}{3,447,294\times 39\%}\right)\times \left(\frac{\frac{43,063}{5years}}{3,447,294}\right)=0.0030\%$$

The probability of a cavity walled dwelling having cavity wall insulation only is taken to be:

$$P\left(Cavity\right)=P\left(Cavity\right)-P\left(Cavity\&Loft\right)=\left(\frac{\frac{80,067}{5years}}{3,447,294\times 39\%}\right)-\left(\left(\frac{\frac{80,067}{5years}}{3,447,294\times 39\%}\right)\times \left(\frac{\frac{43,063}{5years}}{3,447,294}\right)\right)=1.2\%$$

and the probability of a dwelling with cavity walls receiving loft insulation only is:

$$P\left(Loftonly,dwellingswithcavitywalls\right)=P\left(Loft\right)-P\left(Cavity\&Loft\right)=\left(\frac{\frac{43,063}{5years}}{3,447,294}\right)-\left(\left(\frac{\frac{80,067}{5years}}{3,447,294\times 39\%}\right)\times \left(\frac{\frac{43,063}{5years}}{3,447,294}\right)\right)=0.25\%$$

The process was similar for solid-walled dwellings. We estimated the annual probability of solid wall insulation as follows:

$$P\left(SolidWall\&Loft\right)=P\left(Solid\right)xP\left(Loft\right)=\left(\frac{\frac{6,863}{5years}}{3,447,294\times 61\%}\right)\times \left(\frac{\frac{43,063}{5years}}{3,447,294}\right)=0.00016\%$$

$$P\left(SolidWallOnly\right)=P\left(Solid\right)-P\left(Solid\&Loft\right)=\left(\frac{\frac{6,863}{5years}}{3,447,294\times 61\%}\right)-\left(\left(\frac{\frac{6,863}{5years}}{3,447,294\times 61\%}\right)\times \left(\frac{\frac{43,063}{5years}}{3,447,294}\right)\right)=0.065\%$$

$$P\left(Loftonly,dwellingswithsolidwalls\right)=P\left(Loft\right)-P\left(Solid\&Loft\right)=\left(\frac{\frac{43,063}{5years}}{3,447,294}\right)-\left(\left(\frac{\frac{6,863}{5years}}{3,447,294\times 61\%}\right)\times \left(\frac{\frac{43,063}{5years}}{3,447,294}\right)\right)=0.25\%$$

BEIS (2019). Household Energy Efficiency Statistics, detailed report 2019. Department for Business, Energy & Industrial Strategy, London, UK. https://www.gov.uk/government/statistics/household-energy-efficiency-statistics-detailed-report-2019. Accessed October 20th, 2019.

## Footnotes

1 WFPs are a tax-free annual cash payment paid to individuals who receive a State Pension or other benefit to help cover fuel costs.
